# Children's physiological reactivity in emotion contexts and prosocial behavior

**DOI:** 10.1002/brb3.1380

**Published:** 2019-09-15

**Authors:** Brianne R. Coulombe, Kristen L. Rudd, Tuppett M. Yates

**Affiliations:** ^1^ University of California Riverside Riverside California

**Keywords:** autonomic nervous system, emotion elicitation, pre‐ejection period, prosocial behavior, quadratic, respiratory sinus arrhythmia, self‐regulation

## Abstract

**Introduction:**

Building on prior evidence that prosocial behavior is related to the regulation of personal distress in difficult situations, and given that physiological regulation is a central contributor to effective emotion regulation, this investigation evaluated whether and how children's autonomic nervous system (ANS) reactivity during emotion challenges influenced later expressions of prosocial behavior.

**Methods:**

The current study utilized a diverse sample of school‐aged children (*N = *169; 47.9% female; 47.3% Latinx) to evaluate relations between children's parasympathetic (i.e., respiratory sinus arrhythmia; RSA) and sympathetic (i.e., pre‐ejection period; PEP) reactivity in response to each of three film‐elicited emotion challenges (i.e., sadness, happiness, and fear) at age 7 and both observed and parent‐reported prosocial behavior one year later.

**Results:**

Children's parasympathetic reactivity to a film eliciting sadness evidenced a nonlinear relation with later prosocial sharing such that children who evidenced either RSA withdrawal or augmentation in response to the sad emotion challenge engaged in higher levels of prosocial behavior than children who evidenced relatively low or absent reactivity. Parasympathetic reactivity to films eliciting happiness or fear was not significantly related to later prosocial behavior. Likewise, children's sympathetic reactivity in response to the emotion challenges did not significantly predict later prosocial behavior.

**Conclusions:**

These findings provide preliminary support for a nonlinear association between children's parasympathetic emotion reactivity and later prosocial behavior, and suggest that children's ANS regulation in sad emotion contexts may be particularly important for understanding prosocial development.

## Graphical abstract

Building on prior evidence that prosocial behavior is related to the regulation of personal distress in difficult situations, and given that physiological regulation is a central contributor to effective emotion regulation, this investigation evaluated whether and how children's autonomic nervous system (ANS) reactivity during emotion challenges influenced later expressions of prosocial behavior. Children's parasympathetic reactivity to a film eliciting sadness evidenced a nonlinear relation with later prosocial sharing such that children who evidenced either respiratory sinus arrhythmia withdrawal or augmentation in response to the sad emotion challenge engaged in higher levels of prosocial behavior than children who evidenced relatively low or absent reactivity. These findings provide preliminary support for a nonlinear association between children's parasympathetic emotion reactivity and later prosocial behavior, and suggest that children's ANS regulation in sad emotion contexts may be particularly important for understanding prosocial development.

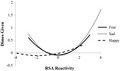

## 1 INTRODUCTION

Over the past two decades, researchers have directed increased attention to three prominent areas of developmental science. First, strength‐based approaches to research have extended the study of development across the adaptive continuum to encompass not only negative outcomes, but also positive ones. For example, studies of prosocial behaviors, or voluntary behaviors intended to benefit others (Batson & Powell, 2003), have joined ongoing efforts to understand problematic and antisocial behaviors. Second, consistent with the framework of developmental psychopathology (Sroufe & Rutter, 1984), researchers have begun to recognize that a shared set of adaptive systems underlies both positive and problematic adaptation. Thus, researchers are increasingly focused on core adaptive processes, such as self‐regulation, which are thought to influence adjustment in multiple domains, over time, and across the adaptive continuum (Eisenberg & Fabes, 1992). Third, scientists have called for multilevel investigations of these core adaptive processes in accord with dynamic systems theories of development (Thelen & Smith, 1998), which hold that relations between elements of a system, in addition to the constituent elements themselves, best account for adaptation (Gottlieb & Halpern, 2002). Thus, studies of core adaptive systems, including self‐regulation, have expanded beyond emotional and behavioral levels of analysis to include physiological indicators of adaptation, particularly the autonomic nervous system (ANS; Gunnar & Vazquez, 2015; Vohs & Baumeister, 2016).

Building on these convergent advances in developmental science, the current investigation evaluated prospective associations between children's ANS reactivity in response to a series of emotion challenges and their prosocial behaviors as observed during a laboratory‐administered donation task and as rated by parents one year later. In doing so, this study addressed several gaps in the fields of social and psychobiological development. First, in contrast to prior studies, which have favored potentially biased self and informant reports of prosocial behavior (Diener & Kim, 2004), this investigation employed an observational laboratory assessment of children's prosocial donating behavior in conjunction with parent reports. Second, relative to the abundance of studies linking self and informant reports of children's emotional and behavioral self‐regulation with prosocial behavior (e.g., Diener & Kim, 2004; Padilla‐Walker & Christensen, 2011), this investigation examined relations between physiological measures of ANS reactivity and prosocial behavior. Third, given the influence of contextual features on the expression and meaning of ANS reactivity (Burt & Obradović, 2013), as well as the relevance of emotion processes in prosocial development (Bandstra, Chambers, McGrath, & Moore, 2011), this study explored relations between prosocial behavior and children's ANS reactivity in response to three emotion contexts tapping sadness, happiness, and fear.

### 1.1 Self‐regulation and prosocial behavior

Self‐regulation encompasses the ability to flexibly modify behavior in response to the biological, cognitive, emotional, and social demands of the environment (Calkins & Fox, 2002; Rueda, Posner, & Rothbart, 2011). Prior studies have documented the role of various self‐regulatory processes in children's prosocial behavior. For example, in an early study of self‐regulation and prosocial behavior, Eisenberg et al. (1996) found that children with high attentional control as reported by mothers, fathers, and teachers also received the highest number of prosocial nominations by their peers. Similar patterns have been reported in studies of toddlers, wherein parent reports of toddlers' self‐regulation skills at the beginning of the preschool semester correlated positively with teacher reports of children's prosocial behavior at the end of the semester (Diener & Kim, 2004). Extending to later childhood, Padilla‐Walker and Christensen (2011) found that self and parent reports of child self‐regulation mediated the relation between a concurrent measure of positive parenting and later parent reports of children's prosocial behaviors. Indeed, self‐regulation has been so robustly connected with children's positive social development that researchers have begun directing intervention efforts toward the promotion of self‐regulation in schools (Flook, Goldberg, Pinger, & Davidson, 2015).

Although a strong body of evidence indicates that multiple aspects of self‐regulation are involved in prosocial behavior (Eisenberg, 2010), only a few studies have examined relations between physiological measures of self‐regulation and prosocial behavior (for a review, see Hastings & Miller, 2014). Given that physiological regulation is a central contributor to effective emotional regulation (Gross, 1998, 2015), and prosocial behavior is related to the way in which children manage their personal distress in difficult situations (Eisenberg et al., 1989), it stands to reason that children's psychobiological regulation, particularly in contexts that elicit negative valence emotions, will influence prosocial development. In support of this assertion, a study of adults found that elevations in cortisol following a public speaking task, which suggests an adaptive mobilization of the neuroendocrine stress response system, were positively related to concurrent observations of prosocial behavior in a sharing paradigm (von Dawans, Fischbacher, Kirschbaum, Fehr, & Heinrichs, 2012). Likewise, in childhood, prior research has shown that physiological regulation during a disappointment task was associated with children's prosocial behavior 6 months later (Scrimgeour, Davis, & Buss, 2016).

Although a multitude of psychobiological systems may be involved in prosocial behavior, the ANS, particularly its parasympathetic branch, may be especially relevant for understanding prosocial actions given its characterization as the social orienting system of the mammalian stress response (Porges, 1995, 2007). The parasympathetic branch of the ANS is responsible for energy preservation and the maintenance of homeostasis (i.e., rest and digest), whereas the sympathetic branch of the ANS is responsible for energy mobilization and action (i.e., fight and flight; Hastings et al., 2008; Porges, 2007). Ideally, these two ANS branches work in conjunction to maintain homeostasis during periods of rest, mobilize an appropriate reaction in response to challenge, and return to homeostasis once a challenge has resolved. Although several measures exist for assessing individual branches of ANS regulation, the cardiac system affords the unique opportunity to examine the distinct contributions of both parasympathetic and sympathetic branches as they operate in tandem to modulate heart rate. In addition, cardiography constitutes a relatively noninvasive method to collect real‐time measures of ANS regulation, as compared to other regulatory markers that operate across an extended time frame (e.g., cortisol responses peak ~15 min after stimuli; Granger et al., 2007).

Given prior suggestions that the inhibitory influence of the parasympathetic nervous system is particularly relevant for modulating social engagement (Miller, Kahle, & Hastings, 2015; Porges, 1995), researchers have begun investigating how cardiac measures of parasympathetic regulation correspond to prosocial behavior. As an index of parasympathetic regulation, respiratory sinus arrhythmia (RSA) reflects vagally mediated variation in heartbeat as a function of respiration, which is thought to promote flexible engagement with social stimuli in the environment (Porges, 2007). At rest, high levels of RSA maintain a calm homeostatic state that features a low resting heart rate and confers a capacity to mobilize an adaptive response in accord with contextual demands. However, the optimal pattern of RSA reactivity in response to environmental challenges will differ based on the nature of the stressor (Hastings, Kahle, & Han, 2014). Indeed, Porges' (1995, 2007) polyvagal theory emphasizes the adaptive significance of flexible augmentation and withdrawal of the parasympathetic system as appropriate to contextual demands. For example, when faced with a surprising or startling stimulus, RSA should decrease to withdraw its inhibitory influence on the sympathetic branch of the ANS, which, in turn, should increase in activation (Kreibig, 2010). However, when children are specifically instructed to modulate their emotional arousal or when an environmental challenge necessitates increased attention, RSA should increase to augment inhibition of the sympathetic branch of the ANS and enhance focused engagement (Davis, Quiñones‐Camacho, & Buss, 2016; Suess, Porges, & Plude, 1994).

Relative to the parasympathetic nervous system, the sympathetic branch of the ANS has received far less attention in studies of self‐regulation and social development. This likely reflects the comparatively greater difficulty of assessing sympathetic regulation, as well as the long‐standing characterization of the parasympathetic system as central to social communication and engagement (Porges, 1995, 2007). In the cardiac system, sympathetic regulation is indicated by the pre‐ejection period (PEP), which is a systolic time interval representing the elapsed duration from the beginning of electrical stimulation until the ejection of blood from the left ventricle (Berntson, Lozano, Chen, & Cacioppo, 2004). In situations that warrant cardiac mobilization, PEP intervals will shorten to facilitate an increase in the number of heart cycles per epoch (i.e., heartbeats). However, in situations that demand attentional engagement, PEP will typically lengthen to support a slow and regulated heart rate.

Although the parasympathetic and sympathetic branches of the ANS work in conjunction to modulate heart rate via RSA and PEP, respectively, the majority of research has examined either parasympathetic or (less often) sympathetic regulation in isolation, and rarely with regard to prosocial behavioral expressions. Theoretical assertions regarding the relation between parasympathetic regulation and prosocial behavior support opposing hypotheses. For example, RSA withdrawal in response to the distress of another person has been posited to reflect an empathic response, which would promote prosocial behavior (Hastings, Zahn‐Waxler, Robinson, Usher, & Bridges, 2000). However, others suggest that RSA withdrawal may reflect self‐oriented feelings of subjective distress, which would interfere with other‐oriented expressions of prosocial behavior (Hastings & Miller, 2014). Empirical studies examining relations between RSA reactivity and prosocial behavior have returned similarly mixed findings. For example, in a study of 2‐year‐old children, Gill and Calkins (2003) found that children who showed RSA withdrawal in response to an empathy‐inducing stimulus (i.e., an audio recording of a child crying) evidenced less empathic responding to the stimulus (e.g., concerned affect, shorter latency to respond) than children who did not evidence parasympathetic withdrawal. Similarly, Beauchaine et al. (2013) found that preschoolers who showed greater RSA withdrawal in response to a difficult block‐building challenge were rated as less prosocial by their mothers than children who evidenced lower RSA withdrawal. In contrast, Cui et al. (2015) found that adolescents who experienced less RSA withdrawal in response to a conversation about an event that made them angry evidenced less self‐reported prosocial behavior than their more reactive peers. Likewise, Liew et al. (2011) found that children who exhibited low levels of RSA withdrawal in response to a fear‐evoking jumping spider toy (i.e., low parasympathetic reactivity) were less prosocial in an observational helping paradigm as compared to their more reactive peers. Indeed, Scrimgeour et al. (2016) found that RSA withdrawal in response to a disappointment task at age 3.5 was positively related to parent reports of prosocial behavior at age 4. Complicating things further, Miller, Kahle, and Hastings (2017) found that children who evidenced RSA augmentation while receiving information about an organization aimed at helping sick children were more likely to donate money to that organization, but other studies have not found significant relations between RSA reactivity and prosocial response tendencies (Eisenberg et al., 1989).

Akin to theories of parasympathetic regulation, researchers have offered opposing theories of sympathetic regulation and prosocial behavior. On the one hand, increased sympathetic arousal may signal personal distress, which motivates self‐oriented rather than other‐oriented (i.e., prosocial) behaviors (Eisenberg, Fabes, Schaller, Carlo, & Miller, 1991). On the other hand, sympathetic arousal may reflect and facilitate engagement with others, which could aid in the mobilization of prosocial responses (Miller, 2018; Zahn‐Waxler, Cole, Welsh, & Fox, 1995). Empirical evidence examining sympathetic involvement in prosocial behavior is limited, but similarly mixed. For example, one study found that girls who evidenced greater skin conductance reactivity while watching a film of two distressed children were rated as less helpful by their mothers (Fabes, Eisenberg, & Eisenbud, 1993). In contrast, a study of adults found that participants who evidenced greater skin conductance reactivity in response to watching someone receive a painful shock were more likely to engage in later prosocial behaviors (Hein, Lamm, Brodbeck, & Singer, 2011). In yet another pattern of results, Beauchaine et al. (2013) did not find a significant relation between PEP reactivity in response to a challenging block‐building task and parent reports of prosocial behavior.

In sum, theory suggests that the ability to flexibly engage with emotional stimuli, particularly negative valence emotions, may promote children's prosocial behavior (Bandstra et al., 2011; Eisenberg et al., 1989). By extension, we hypothesized that ANS regulation in response to emotionally challenging film clips would be associated with children's later prosocial behavior. However, the contradictory evidence regarding the role of parasympathetic regulation in this process and the limited research examining the role of sympathetic regulation highlight the need for further studies to elucidate the nature of these relations and explore factors that may contribute to the extant state of confusion in the field.

### 1.2 Reconciling conflicting evidence: A nonlinear hypothesis

Efforts to reconcile conflicting evidence regarding the relation between ANS regulation and prosocial behavior are ongoing. One hypothesis is that the nature of this association may be most accurately modeled using a nonlinear function. For example, Eisenberg (2010) suggests that empathy (and perhaps prosocial behavior by extension) requires some level of physiological arousal, yet a surplus of arousal in response to empathy‐inducing stimuli may be associated with personal distress. In turn, the self‐focused nature of personal distress may undermine prosocial expressions by motivating actions to alleviate one's own distress rather than the distress of another person. In support of this hypothesis, personal distress has been associated with higher levels of physiological arousal (e.g., skin conductance, heart rate) than sympathy (Eisenberg & Fabes, 1990) and is typically negatively related to prosocial expressions (for a review, see Eisenberg & Eggum, 2009). On the other end of the spectrum, low physiological arousal in response to challenge, particularly in emotion‐eliciting contexts, has been related to callous unemotionality, which is characterized by low affective empathy and, presumably, less prosocial behavior (Anastassiou‐Hadjicharalambous & Warden, 2008).

Prior theory and research point to the potential for complex, curvilinear relations between ANS regulation and prosocial behavior such that either extreme or muted patterns of reactivity may be negatively related to prosocial behavior. In support of this hypothesis, Clark, Skowron, Giuliano, and Fisher (2016) found that children's baseline RSA evidenced a negative quadratic association with concurrent parent reports of prosocial behavior. Extending over time, Miller et al. (2017) found that children with moderate levels of baseline RSA evidenced greater self‐reported prosocial behavior and empathic concern (e.g., a hug, kind words) in response to the feigned injury of an examiner concurrently, as well as higher levels of teacher‐, parent‐, and self‐reported prosocial behavior five years later, as compared to children with either very high or very low baseline RSA levels. These associations are also apparent in studies of observed prosocial behavior. For example, Zhang and Wang (2019) found that moderate baseline RSA predicted greater levels of prosocial sharing in childhood as compared to either low or high levels of baseline RSA.

Although several studies have documented quadratic associations between baseline RSA and prosocial behavior, very few have examined nonlinear relations between physiological *reactivity* to environmental challenges and prosocial behavior. Kogan et al. (2014) documented a negative quadratic relation between RSA activity during film clips of a person in distress and adults' self‐reported prosocial behaviors such that moderate, but not extreme, levels of RSA activity during the film were positively associated with self‐reported prosocial behavior, but this study did not examine *reactivity* in terms of the residualized *change* from baseline to challenge (El‐Sheikh, Harger, & Whitson, 2001; Manuck, Kasprowicz, & Muldoon, 1990; Rudd & Yates, 2018). Likewise, a recent study showed that patterns of RSA across baseline and film conditions predicted children's helping behaviors (Miller, Nuselovici, & Hastings, 2016), but this study did not examine the magnitude of RSA response from baseline to challenge (i.e., reactivity). Although no prior studies of prosocial behavior have examined PEP reactivity, extreme sympathetic arousal may connote subjective distress that impedes prosocial engagement, whereas the absence of sympathetic mobilization may connote a low motivation to respond to the needs of others (i.e., a negative quadratic relation; Miller, 2018). This study advanced the current literature by examining relations between children's ANS *reactivity* to emotion challenges and prosocial behaviors one year later. Moreover, we examined both parasympathetic reactivity and sympathetic reactivity as related both observed and parent‐reported expressions of prosocial behavior.

### 1.3 Reconciling conflicting evidence: A contextual hypothesis

Given that effective self‐regulation is characterized by the ability to modify behavior in response to situational demands (Rueda et al., 2011), it is important to consider the contextual features of a given challenge when seeking to understand apparent discrepancies in studies of ANS regulation and development (for review, see Burt & Obradović, 2013). For example, as described earlier, both the direction and the intensity of ANS reactivity may vary across challenges (e.g., a startling or frightening stimulus vs. one that requires a calm and attentive state of arousal; Krantz & Manuck, 1984; Suess et al., 1994). Likewise, the relative salience of parasympathetic versus sympathetic activity may vary by context (e.g., a stressful social stimulus vs. a stimulating taste challenge; Alkon et al., 2003). Finally, other data suggest that the adaptive implications of ANS regulation may vary depending on whether or not another person is present during the challenge (e.g., a challenging puzzle completed with or without a caregiver present; Skowron, Cipriano‐Essel, Gatzke‐Kopp, Teti, & Ammerman, 2014).

As a central motivator and modifier of human behavior (Deci, 1996; Easterbrook, 1959), emotion is likely to be a key contextual factor that influences ANS regulation (Ekman, Levenson, & Friesen, 1983) and social behavior (Lench, Flores, & Bench, 2011). For example, Eisenberg et al. (1988) found that children evidenced increased heart rate, which reflects the combined influence of parasympathetic and sympathetic reactivity, in response to a film clip designed to evoke anxiety, but decreased heart rate in response to a film clip designed to evoke sadness (Eisenberg et al., 1989). Behaviorally, children who reported feeling sad (as opposed to happy or distressed) after watching a film clip about children in the hospital were more likely to express interest in helping those sick children with their homework (Eisenberg et al., 1989). Likewise, in a study of children's behavioral responses to an examiner's distress, Bandstra et al. (2011) found that children were more likely to express helping behaviors, such as comfort or distracting with a toy, when the examiner feigned sadness rather than pain. Building on prior studies that point to the salience of emotion for understanding patterns of ANS regulation and social behavior, this investigation explored children's parasympathetic and sympathetic reactivity in response to a series of film clips designed to elicit sad, happy, or fearful emotions as related to both observed and parent‐reported expressions of prosocial behavior one year later.

### 1.4 The current study

Recent evidence suggests that self‐regulation in the context of emotional arousal is related to both problematic and positive social behaviors, including prosocial actions (for a review, see Eisenberg, 2010). Relative to studies examining relations of prosocial behavior with emotional, cognitive, and/or behavioral capacities for self‐regulation (Eisenberg et al., 1996; Flook et al., 2015), less is known about whether and how physiological reactivity may relate to prosocial behavior. Thus, this study sought to advance our understanding of ANS reactivity and prosocial behavior by evaluating both linear and nonlinear models of association between 7‐year‐old children's parasympathetic and sympathetic reactivity in response to sad, happy, and fearful emotion films and both observed and parent‐reported expressions of prosocial behavior one year later.

The current study drew on a large and diverse school‐aged sample to test hypotheses regarding how children's parasympathetic and sympathetic reactivity in response to emotion‐eliciting films would be related to later prosocial behaviors. Specifically, we hypothesized that the nature of these relations would be nonlinear such that moderate levels of ANS reactivity would be positively associated with prosocial behavior, but both extreme reactivity, which may signal subjective distress (Eisenberg & Eggum, 2009), and muted or absent reactivity, which may signal callous unemotionality (Anastassiou‐Hadjicharalambous & Warden, 2008), would be negatively related to prosocial behavior. Given the relative dearth of studies examining the role of sympathetic involvement in prosocial behavior, and theoretical assertions that the parasympathetic nervous system might be particularly relevant for understanding social communication and engagement (Porges, 2007), we hypothesized that parasympathetic reactivity would be more strongly related to prosocial behavior than sympathetic reactivity. Further, we expected that physiological reactivity in response to negative valence emotions, particularly sadness, would be most robustly related to prosocial expressions, in light of prior studies demonstrating that sad emotion contexts may be especially relevant for understanding prosocial responding (Bandstra et al., 2011; Eisenberg et al., 1989). Finally, given prior evidence that prosocial behavior may vary by gender (e.g., Fabes et al., 1993; Veenstra et al., 2008), race/ethnicity (e.g., Carlo, Roesch, Knight, & Koller, 2001), and/or socioeconomic status (SES; e.g., Benenson, Pascoe, & Radmore, 2007), we held these covariates constant in the current analyses. Further, because the emotion‐eliciting film clips were dependent on children's ability to understand the film content, we also included children's verbal ability as a covariate.

## 2 METHOD

### 2.1 Participants

The current sample was drawn from an ongoing study of development among 250 caregiver–child dyads. Participants in these analyses (*N* = 169; 47.9% female) completed a laboratory assessment of ANS reactivity during a series of emotion‐eliciting films at age 7 (*M*_age_ = 7.12 years, *SD* = 0.22). An additional seven children did not have valid physiological data due to computer or electrode placement errors, and 22 children were omitted from these analyses because >25% of the obtained cardiac data were not scorable. Children who provided valid physiological regulation data at age 7 (*N* = 169) did not differ from those who did not (*N = *29) in terms of gender, race/ethnicity, family SES, or prosocial behavior. The children in the current sample were diverse with regard to race/ethnicity (11.2% White, 16.6% Black, 47.3% Latinx, 24.9% multiracial) and representative of the southern California community from which they were recruited (U.S. Census Bureau, 2011). Participating caregivers were biological mothers (93.2%), female extended kin (3.0%), foster/adoptive mothers (2.6%), stepmothers (0.5%), and biological fathers (0.5%). The average family SES score, based on the Hollingshead (1975) Four‐Factor Index of Social Status, was 32.19 (*SD* = 12.24), which corresponds to semi‐skilled employment (e.g., sales clerk). At age 8, 162 families (95.86%) completed a follow‐up assessment, which included both an observational and parent‐reported measurements of prosocial behavior. There were no significant differences between dyads who completed both visits and those who did not on all study variables.

### 2.2 Procedure

Caregivers were recruited to participate in a longitudinal study of children's early learning and development via flyers placed in community‐based preschool programs and child development centers. Exclusionary criteria included children with diagnosed developmental disabilities or delays (*n* = 3), children who were unable to understand English (*n* = 4), and children outside the recruitment age range of 45–54 months (not tracked). At each data wave, dyads completed an extensive laboratory assessment that included both observational and survey‐based measures of regulation and adaptation with the child and the primary caregiver. Caregivers were compensated with $25/hr of assessment, and children received a small gift after each visit. Informed consent and assent were obtained from the child's legal guardian and the child, respectively. All procedures were approved by the human research review board of the participating university.

### 2.3 Measures

#### 2.3.1 ANS reactivity

At age 7, caregiver–child dyads were told that they would be watching a series of film clips beginning with a film about the outdoors, followed by clips about (a) a family, (b) a dinner, and (c) a train, which were adopted from prior work (Bennett & Lewis, 2011). ANS activity was assessed during each film clip using four spot electrodes placed on the neck and torso to collect impedance and respiratory measures, and three spot electrodes placed on the right clavicle, left lower rib, and right abdomen for electrocardiogram (ECG) measures. RSA and PEP data were extracted and scored using Mindware's 3.0.10 analysis program (http://www.mindwaretech.com). RSA data were filtered, extracted, and scored utilizing the Mindware software algorithm to calculate the differences in interbeat intervals (i.e., the distance between the R waves between beats) on the ECG reading, and respiratory rates were derived from the d*Z*/d*t* signal. PEP data were obtained using d*Z*/d*t* waveforms to quantify the time interval in milliseconds from the onset of the ECG Q‐wave to the B point of the d*Z*/d*t* wave (Berntson et al., 2004). Data cleaning procedures included screening for outliers (i.e., >3*SD*; Alkon, Boyce, Davis, & Eskenazi, 2011) minute‐by‐minute in relation to each child's data pattern.

Baseline values for both RSA and PEP were indicated by the average of six 30‐s epochs across a 3‐min film baseline during which children viewed a neutral nature scene. ANS reactivity was indicated by standardized residual values obtained from a regression of the average across four epochs during each 2‐min emotion‐eliciting film on resting RSA/PEP values to yield an index of each child's relative change in RSA/PEP from baseline to challenge as compared to other children in the sample (El‐Sheikh et al., 2001; Manuck et al., 1990; Rudd & Yates, 2018). The resultant scores captured the child's ANS reactivity to (a) a sad scene depicting three young children sobbing after they learn that their mother has died from *Crooklyn*, (b) a happy scene depicting a children's food fight from *Hook*, and (c) a scary/fear scene depicting a train barreling down on two children from *Stand By Me*. Higher standardized residual scores indicated RSA augmentation (i.e., parasympathetic activation) and PEP elongation (i.e., sympathetic withdrawal) in response to the film clips, whereas lower standardized residual scores indicated RSA withdrawal and PEP attenuation. Clips were administered in a standardized order—sad, happy, fear—with 1‐min neutral nature film clips separating each emotion elicitation. We used the initial neutral baseline film for all reactivity calculations because the intervening nature clips also encompassed recovery processes and thus were not true baselines.

#### 2.3.2 Prosocial behavior

##### Observational measure

At age 8, children's prosocial donating behavior was assessed in a donation task that was adapted from Grusec and Redler (1980). First, after completing a difficult memory assessment, children received a prize of ten dimes “because they tried their best.” Second, the examiner laid the ten dimes on the table in front of the child in a horizontal line, and then explained that the research team was “collecting money to support local kids who were really sick.” Third, the examiner pointed to a labeled jar that contained several coins and explained that the child could put some of the prize money into the jar if they wanted. The examiner then left the room for one minute to retrieve something, and the child was left alone to decide how many dimes to donate. Although most children made their donation during this time period, children were able to make a donation at any point during the remainder of the visit (e.g., some children put in a few dimes right away, but then put in another few dimes later in the visit). Prosocial sharing was indicated by the total number of dimes the child placed in the jar by the end of the visit.

##### Parent reported measure

At ages 7 and 8, parents rated their child's prosocial behavior using the Strengths and Difficulties Questionnaire (SDQ; Goodman, 1997). The SDQ is a well‐validated measure of children's psychopathology and social behaviors. Parents rated their child's prosocial behavior on five items (e.g., your child is helpful if someone is hurt, upset, or feeling ill) using a 5‐point Likert scale ranging from *never* (1) to *almost always* (5) at each time point (*α*_age 7_ = 0.692; *α*_age 8_ = 0.773). In the current study, items from the SDQ were integrated into a broader survey containing behavioral items from several measures, which used the aforementioned 5‐point response scale instead of the SDQ's original 3‐point scale (i.e., *not true, somewhat true, certainly true*).

#### 2.3.3 Verbal ability

At age 7, children completed the Letter‐Word subtest from the Woodcock Johnson III Tests of Achievement (WJ‐III; Woodcock, Mather, & McGrew, 2001). The WJ‐III is a well‐validated measure of academic achievement designed for use from age 2 to adulthood. In the Letter‐Word subtest, children were asked to read a series of increasingly difficult words out loud beginning with a six‐item basal level and continuing until six consecutive items were missed. The Letter‐Word standard score (*M* = 111.24, *SD* = 14.34) was included in all analyses as a proxy for verbal ability, which is known to be associated with information processing (Neuhaus, Foorman, Francis, & Carlson, 2001) and prosociality (Miles & Stipek, 2006).

#### 2.3.4 Data preparation and analytic plan

All analyses were performed using the lavaan package in RStudio (Rosseel, 2012). Standardized residual scores were computed to assess the extent to which children's ANS reactivity during each emotion‐elicitation film deviated from the sample regression line. Data were examined for non‐normality to render parametric statistics valid (Afifi, Kotlerman, Ettner, & Cowan, 2007). Observational measures of prosocial donating behavior were missing for 26 (12.3%) children, either because they did not complete the age 8 assessment in person (*n* = 10; 4.7%), or because the task was not administered due to its delayed addition to the assessment battery (*n* = 16; 7.6%). Seven children (4.14%) were missing parent reports of prosocial behavior at age 8 because they did not complete the age 8 assessment. One child (0.01%) was missing verbal ability data due to an examiner administration error. All study variables were mean‐centered and standardized to reduce multicollinearity and allow for more direct comparisons between the observational and parent‐reported measures. Missing data were addressed using the full information maximum‐likelihood procedure in RStudio.

A multivariate analysis of variance (MANOVA) evaluated differences in study variables as a function of children's gender, race/ethnicity, and their interaction. Correlational analyses evaluated bivariate relations between study variables. Separate polynomial regression models tested the relation of children's RSA and PEP reactivity in response to each film clip with their later observed and parent‐reported prosocial behavior while holding child gender, race/ethnicity, verbal ability, family SES, and prosocial behavior (available only for parent reports) constant.

## 3 RESULTS

### 3.1 Preliminary analyses

Repeated‐measures analyses of variance (ANOVA) followed by post hoc *t* tests evaluated the effectiveness of the film‐based emotion elicitations and the postemotion neutral films to elicit ANS reactivity and potential carryover effects, respectively, in accordance with prior work (Bush, Alkon, Obradović, Stamperdahl, & Boyce, 2011). There were significant differences in RSA across the emotion film challenges and postemotion recovery periods (Wilks' *λ* = 0.884, *p* = .002). Follow‐up *t* tests revealed a significant increase in RSA from baseline to each emotion film (*t*_sad_ = −4.222, *p* < .001, *t*_happy_ = −2.581, *p* = .011, *t*_fear_ = −3.038, *p* = .003). There was a significant decrease in RSA from the sad film to the postemotion neutral film (*t*_sad_ = 2.771, *p* = .006), a nonsignificant decrease in RSA from the happy film to the postemotion neutral film (*t*_happy_ = 0.159, *p* = .874), and a marginal decrease in RSA from the fear film to the postemotion neutral film (*t*_fear_ = 1.776, *p* = .077). Importantly, there were no significant differences in RSA between baseline and neutral films following the sad (*t*_sad_ = −1.347, *p* = .180) and fear emotion conditions (*t*_fear_ = −0.702, *p* = .484), though the recovery following the happy film was incomplete (*t*_happy_ = −2.262, *p* = .025). There were no significant differences in PEP across the emotion films and recovery periods (Wilks’ *λ* = 0.951, *p* = .218).

### 3.2 Descriptive and bivariate analyses

Descriptive statistics and bivariate correlations for study variables are reported in Table 1. A MANOVA revealed no significant main effects of child gender (Wilks’ *λ* = 0.946, *p* = .110) race/ethnicity (Wilks’ *λ* = 0.959, *p* = .230), or their interaction (Wilks’ *λ* = 0.977, *p* = .577) across study variables. Bivariate correlations indicated that family SES was positively related to children's verbal ability and baseline PEP. Verbal ability was positively related to observed prosocial donating behavior. Baseline RSA was positively associated with parent‐reported prosocial behavior at age 7, with RSA levels during each emotion film, and with PEP during the sad film. Baseline PEP was positive associated with PEP levels across all emotion films, as well as with RSA during the happy film. RSA during the sad film was positively related to RSA during the happy and scary films. PEP during the sad film was positively related to PEP during the happy and scary films, and PEP during the happy film was positively related to PEP during the scary film. RSA reactivity to fear was positively correlated with PEP reactivity to fear. Finally, parent‐reported prosocial behavior at age 7 was positively correlated with parent‐reported prosocial behavior at age 8.

**Table 1:** Descriptive statistics and correlations among study variables

Study variables	*M* (*SD*)	1	2	3	4	5	6	7	8	9	10	11	12
1. Family SES	31.67 (12.742)	–	–	–	–	–	–	–	–	–	–	–	–
2. Verbal ability (age 7)	111.14 (14.303)	.216**	–	–	–	–	–	–	–	–	–	–	–
3. RSA Baseline – Neutral (age 7)	7.311 (0.903)	.088	.066	–	–	–	–	–	–	–	–	–	–
4. PEP baseline – neutral (age 7)	102.261 (8.196)	.164*	−.122	.219**	–	–	–	–	–	–	–	–	–
5. RSA challenge – sad (age 7)	7.428 (0.954)	.065	.077	.774**	.081	–	–	–	–	–	–	–	–
6. PEP challenge – sad (age 7)	102.819 (9.920)	.062	−.004	.153*	.813*	−.016	–	–	–	–	–	–	–
7. RSA challenge – happy (age 7)	7.355 (0.967)	.060	−.013	.756**	.175*	.845**	.076	–	–	–	–	–	–
8. PEP challenge – happy(age 7)	102.987 (9.345)	−.020	.042	.032	.493**	.059	.457**	.017	–	–	–	–	–
9. RSA challenge – fear (age 7)	7.371 (0.886)	−.011	−.013	.756**	.122	.822**	.058	.823	.069	–	–	–	–
10. PEP challenge – fear(age 7)	102.563 (8.482)	−.073	.078	.146	.657**	.045	.745**	.069	.789**	.140	–	–	–
11. Parent‐reported prosocial behavior (age 7)	3.302 (0.592)	−.093	−.084	.187*	−.039	.134	−.060	.146	−.036	.131	−.059	–	–
12. Parent‐reported prosocial behavior (age 8)	3.355 (0.615)	−.114	−.028	.135	−.049	.139	−.088	.120	−.030	.096	.017	.540**	–
13. Observed prosocial donation behavior (age 8)	2.908 (3.701)	.093	.187*	−.152	−.202	.013	−.218**	.008	−.048	.041	−.135	.079	.071

Note

Challenge scores reflect average RSA/PEP values across each film clip.

Abbreviations: RSA, respiratory sinus arrhythmia; PEP, pre‐ejection period; SES, socioeconomic status.

*p < .05; **p < .01.

### 3.3 Regression analyses

Polynomial regression analyses evaluated linear and quadratic relations between children's ANS reactivity (i.e., RSA or PEP) in response to each emotion film at age 7 (i.e., sad, happy, fearful) and observations of children's prosocial donation behavior at age 8. A second set of regressions evaluated these same relations with parent reports of children's prosocial behaviors at age 8 over and above prior reports at age 7. Following the recommendations of Laird and De Los Reyes (2013), we evaluated each polynomial regression at one order higher in magnitude than that of interest to ensure that the final model adequately captured the nature of the nonlinear relation. Therefore, regressions with quadratic terms were interpreted, only after confirming that all cubic ANS reactivity effects were not significant.

**Figure 1:**
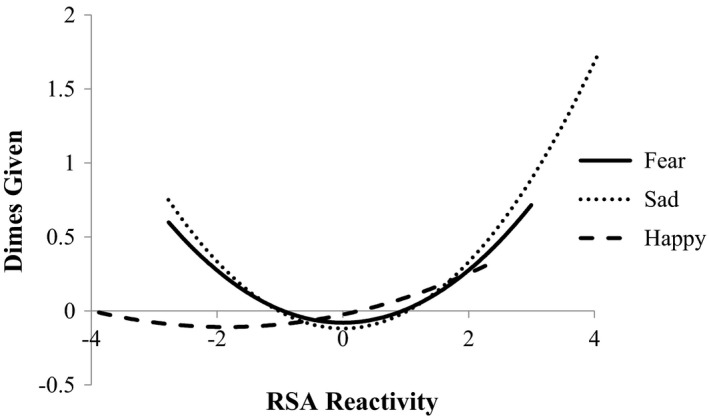
Regression of standardized prosocial donating behavior on RSA reactivity across emotion contexts

Regression analyses predicting observed prosocial donating behavior revealed a significant and positive quadratic, but not linear, effect of children's RSA reactivity to the sad emotion elicitation, but no significant relations with RSA reactivity to either the happy or fear film clips (Table 2). Children who evidenced parasympathetic reactivity via either withdrawal (i.e., low residual scores) or augmentation (i.e., high residual scores) in response to the sad film clip evidenced greater prosocial donating behavior than children who displayed relatively muted or absent levels of RSA reactivity in either direction (i.e., withdrawal or augmentation). As shown in Figure 1, there was no significant relation, quadratic nor linear, between children's RSA reactivity to the happy and fear emotion elicitations and later prosocial behavior. Likewise, there were no significant relations between PEP and later observations of children's prosocial donation behavior (Table 3).

**Table 2:** Regression of prosocial donating behavior on parasympathetic reactivity to sad, happy, and fear emotion‐eliciting film clips

Predictor	SAD				HAPPY				FEAR			
Observation *Parent report*	*B*	*SE*	*z*‐value	*p*	*B*	*SE*	*z*‐value	*p*	*B*	*SE*	*z*‐value	*p*
Gender (female = 1)	0.312	0.159	1.962	.050	0.296	0.163	1.817	.069	0.302	0.163	1.852	.064
	*0.258*	*0.132*	*1.962*	*.050*	*0.248*	*0.133*	*1.871*	*.061*	*0.248*	*0.080*	*1.870*	*.061*
Race (Latinx = 1)	−0.040	0.163	−0.248	.804	−0.070	0.169	−0.417	.676	−0.071	0.168	−0.426	.670
	*0.031*	*0.134*	*0.230*	*.818*	*0.006*	*0.137*	*0.047*	*.963*	*0.008*	*0.083*	*0.055*	*.956*
SES	0.008	0.006	1.248	.212	0.004	0.007	0.656	.512	0.003	0.007	0.378	.706
	*−0.003*	*0.005*	*−0.638*	*.523*	*−0.005*	*0.005*	*−0.965*	.*334*	*−0.006*	*0.005*	*−1.096*	.*273*
Verbal ability	0.008	0.006	1.404	.160	0.010	0.006	1.766	.077	0.010	0.006	1.785	.074
	*0.001*	*0.005*	*0.118*	*.906*	*0.001*	*0.005*	*0.300*	.*764*	*0.001*	*0.005*	*0.313*	*.754*
Prior prosocial behavior	–	–	–	–	–	–	–	–	–	–	–	–
	*0.516*	*0.066*	*7.831*	*<.001*	*0.518*	*0.066*	*7.799*	*<.001*	*0.524*	*0.067*	*7.862*	*<.001*
RSA	0.005	0.080	0.067	.947	0.097	0.085	1.143	.253	0.017	0.087	0.191	.848
	*−0.005*	*0.066*	*−0.074*	*.941*	*−0.027*	*0.070*	*−0.385*	*.700*	*−0.051*	*0.069*	*−0.740*	*.460*
RSA^2^	0.116	0.039	2.946	.003	0.014	0.044	0.316	.752	0.080	0.060	1.324	.186
	*0.059*	*0.044*	*1.735*	.*083*	*−0.002*	*0.037*	−*0.067*	.*947*	*0.034*	*0.039*	*0.868*	.*385*

The values in Roman refer to our observed outcome and those that are italicized refer to the parent reported outcome.

Abbreviations: RSA, respiratory sinus arrhythmia; SES, socioeconomic status.

**Table 3:** Regression of prosocial donating behavior on sympathetic reactivity to sad, happy, and fear emotion‐eliciting film clips

Predictor	SAD				HAPPY				FEAR			
Observation *Parent report*	*B*	*SE*	*z*‐value	*p*	*B*	*SE*	*z*‐value	*p*	*B*	*SE*	*z*‐value	*p*
Gender (female = 1)	0.259	0.165	1.565	.118	0.264	0.162	1.628	.103	0.280	0.162	0.725	.085
	*0.254*	*0.134*	*1.962*	*.131*	*0.245*	*0.133*	*1.871*	*.061*	*0.233*	*0.132*	*1.759*	*.078*
Race (Latinx = 1)	−0.053	0.169	−0.315	.753	−0.113	0.166	−0.677	.498	−0.082	0.166	−0.495	.621
	*0.006*	*0.137*	*0.230*	*.818*	*0.008*	*0.136*	*0.047*	.*963*	*0.029*	*0.134*	*0.220*	*.826*
SES	0.004	0.007	0.577	.564	0.004	0.006	0.621	.534	0.002	0.007	0.311	.756
	*−0.005*	*0.005*	*−0.638*	.*523*	*−0.005*	*0.005*	*−0.973*	*.331*	*−0.003*	*0.005*	*−0.570*	*.569*
Verbal ability	0.011	0.006	1.906	.057	0.011	0.006	1.911	.056	0.013	0.006	2.141	.032
	*0.002*	*0.005*	*0.118*	*.906*	*0.002*	*0.005*	*0.382*	*.703*	*0.000*	*0.005*	*0.082*	*.935*
Prior prosocial behavior	–	–	–	–	–	–	–	–	–	–	–	–
	*0.520*	*0.067*	*7.831*	*.001*	*0.515*	*0.067*	*7.722*	*<.001*	*0.523*	*0.066*	*7.928*	*<.001*
PEP	0.097	0.093	−1.403	.297	−0.276	0.170	−1.622	.105	−0.209	0.139	−1.618	.106
	*0.020*	*0.078*	*−0.074*	*.941*	*−0.020*	*0.076*	−.*267*	.*789*	*0.143*	*0.86*	*1.663*	*.096*
PEP^2^	0.005	0.022	0.217	.828	−0.034	0.048	−0.696	.487	−0.018	0.029	−0.607	.544
	*0.012*	*0.019*	*1.735*	*.083*	*0.006*	*0.016*	*0.347*	*.728*	*0.025*	*0.020*	*1.264*	*.206*

The values in Roman refer to our observed outcome and those that are italicized refer to the parent reported outcome.

Abbreviations: PEP, pre‐ejection period; SES, socioeconomic status.

**Figure 2:**
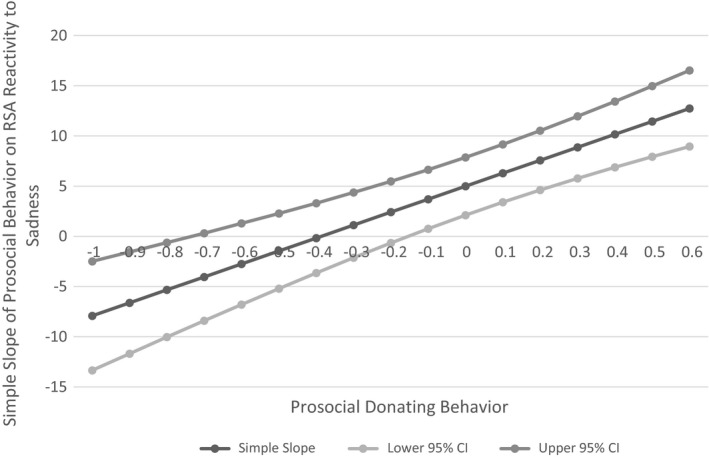
Johnson‐Neyman plot of the region of significance for the simple slope of prosocial donating behavior on RSA reactivity to sadness

We applied the Johnson‐Neyman technique (Johnson & Neyman, 1936; Miller, Stromeyer, & Schwieterman, 2013) to probe the nature of our quadratic RSA sad reactivity effect in the model predicting observed prosocial donation behavior. In contrast to traditional “pick‐a‐point” probing at plus or minus one standard deviation around the average value of the predictor, this technique identifies a “region of significance” at which the simple slope becomes statistically significant and specifies confidence bands that connote the precision of the simple slope estimate (see Bauer & Curran, 2005; Miller et al., 2013, for discussion). As shown in Figure 2, the simple slope of the line tangent to the curve became significant and negative when RSA reactivity values fell below −0.75, but significant and positive when RSA reactivity values rose above 1.09.

**Figure 3:**
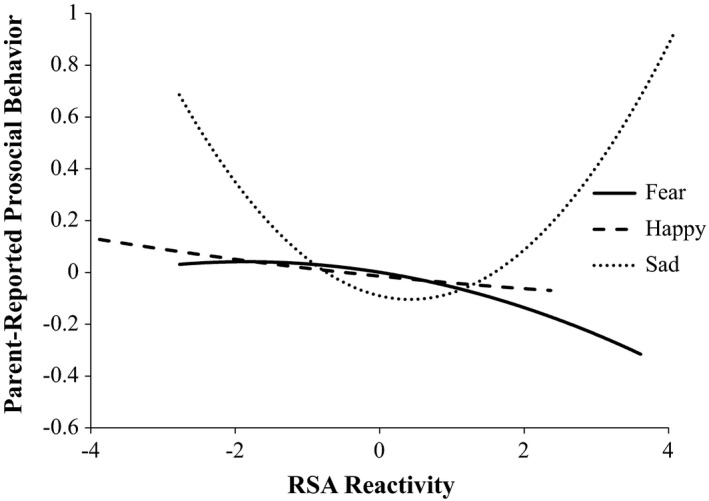
Regression of standardized parent‐reported prosocial behavior on RSA reactivity across emotion contexts

Regression analyses predicting parent‐reported prosocial behavior at age 8 over and above prior parent reports evidenced a similar pattern of findings with a marginal positive quadratic, but not linear, relation between RSA reactivity to the sad emotion elicitation and increased parent‐reported prosocial behavior (Table 2). However, there were no significant relations with RSA reactivity to the happy and fear film conditions (Figure 3). There were no significant relations between PEP and parent‐reported prosocial behavior.

Although the MANOVA did not reveal a main effect of gender, gender emerged as a marginally significant predictor of prosocial behavior in several models, which revealed a trend for girls to evidence more prosocial behavior than boys. Across models, child ethnicity‐race, verbal ability, and family SES did not account for significant variance in prosocial outcomes.

## 4 DISCUSSION

This study demonstrated a significant positive quadratic relation between children's parasympathetic reactivity during an emotion film that elicited sadness and their prosocial donating behavior one year later. Further, these patterns largely replicated when predicting parent‐reported prosocial behavior at age 8 over and above prior prosocial ratings at age 7. In contrast, neither RSA reactivity to film clips designed to elicit happy or fear emotions nor PEP reactivity in response to any of the three emotion film challenges predicted children's later prosocial behaviors. These findings provide empirical support for the theoretical proposition that optimal patterns of ANS reactivity to promote prosocial engagement may be nonlinear (Hastings & Miller, 2014), and extend previous tests of this nonlinear hypothesis in adult samples (Kogan et al., 2014) to young children. Further, the obtained findings are consistent with prior assertions that physiological reactivity during sad emotion contexts may be particularly salient for understanding prosocial engagement (Bandstra et al., 2011; Eisenberg et al., 1989), though remain suggestive given the absence of a counterbalanced stimulus presentation in this investigation.

In line with Porges' (2007) assertion that the parasympathetic branch of the ANS drives the mammalian social engagement system, and consistent with prior studies that point to the relevance of empathy‐inducing stimuli (i.e., sadness; Eisenberg et al., 1989) for understanding prosocial behavior, parasympathetic reactivity to sadness emerged as a significant predictor of prosocial behavior. As in prior studies (Beauchaine et al., 2013; Gill & Calkins, 2003; Miller et al., 2015), the children in this sample generally evidenced a pattern of RSA augmentation and PEP elongation in response to these emotional film stimuli. However, a sizable minority of the children evidenced RSA withdrawal (*n* = 76, 38.58%) and PEP attenuation (*n = *82; 48.52%). Interestingly, prosocial behavior increased with both parasympathetic augmentation and withdrawal, whereas children who failed to mobilize a parasympathetic response to the sad emotion challenge evidenced lower levels of prosocial behavior at follow‐up. Although the positive direction of the obtained quadratic relation between RSA reactivity and prosocial behavior was initially surprising, further consideration of the results suggested two potential interpretations.

First, these findings may indicate that the capacity to engage a parasympathetic response, rather than the specific direction of response, is positively associated with prosocial behavior. Although this dynamic range hypothesis is typically discussed in studies with challenges that precipitate RSA withdrawal (Hinnant & El‐Sheikh, 2009; Staton, El‐Sheikh, & Buckhalt, 2009), it offers a viable explanation of the obtained data wherein children who evidenced more parasympathetic change in response to the sad emotion film also displayed significantly greater levels of prosocial behavior at follow‐up. That said, it is important to consider that there may be an optimal dynamic range of parasympathetic regulation, such that extreme parasympathetic responses in either direction may, ultimately, compromise social adaptation. Given prior suggestions that excessive arousal may undermine other‐oriented social engagement (e.g., prosocial behavior; Eisenberg & Eggum, 2009; Eisenberg et al., 1989), future studies using more extreme emotion challenges are needed to fully evaluate this dynamic range hypothesis.

**Figure 4:**
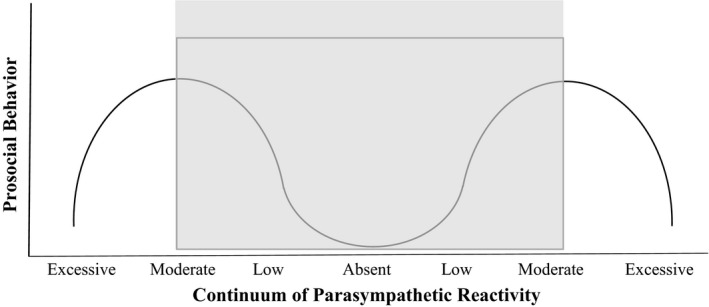
Conceptual interpretation of the observed findings

Consistent with the idea of an optimal dynamic range of response, a second interpretation of these findings is that the range of reactivity responses obtained in this study captured only a portion of the underlying curvilinear relation between parasympathetic reactivity and prosocial responding. As reviewed earlier, prior theory and research suggest that prosocial behavior may be engendered by a moderate range of arousal, such that those who are neither over‐ nor underaroused by the needs of others are most likely to behave prosocially (Eisenberg et al., 1989; Kogan et al., 2014). Although film‐based emotion evocations are commonly used and have demonstrated ecological validity (Gross & Levenson, 1995; Ray, 2007; Uhrig et al., 2016), it is likely that the current paradigm posed a relatively modest regulatory challenge as compared to an in vivo, active challenge involving more intense emotional content with real‐life actors. Paired with a dynamic range hypothesis, which emphasizes the *capacity* to engage a regulatory response more than the *direction* of response, these findings point to complex relations between RSA reactivity and prosocial behavior, only a portion of which may have been captured by the current stimuli (see Figure 4 for a conceptual depiction of this interpretation). In this view, moderate RSA augmentation, which is indicative of focused engagement (Miller et al., 2017), or withdrawal, which is indicative of an empathic response (Hastings et al., 2000), would support prosocial behavior. However, either extreme levels of parasympathetic withdrawal, which may reflect self‐oriented subjective distress (Hastings & Miller, 2014), or extreme levels of parasympathetic augmentation, which may connote an excessively engaged or perseverative response (Buss, Davis, Ram, & Coccia, 2018; Porges, 2007), would undermine prosocial behavior.

### 4.1 Strengths and limitations

The current study tested nonlinear relations of children's parasympathetic and sympathetic reactivity during sad, happy, and fearful emotion‐elicitation films with observed and parent‐reported measures of prosocial behavior one year later. The obtained results supported prior assertions that (a) parasympathetic reactivity may be more relevant for understanding prosocial behavior than sympathetic reactivity (Porges, 1995, 2007), (b) the nature of these relations may be nonlinear (Kogan et al., 2014; Miller et al., 2017), and (c) empathy‐inducing stimuli, such as sadness, may be more relevant than other emotion contexts for understanding prosocial development (Eisenberg, 2010; Eisenberg & Fabes, 1990). Although recent studies have considered nonlinear relations between ANS regulation and adjustment outcomes, including prosocial behavior (for a review, see Hastings & Miller, 2014), prior research has focused on baseline RSA, rather than reactivity (e.g., Clark et al., 2016; Zhang & Wang, 2019), and no study to our knowledge has evaluated relations between PEP and prosocial behavior. Despite advancing our understanding of ANS reactivity and prosocial behavior, several limitations necessarily qualify the interpretation of the current findings.

First, the emotion‐elicitation film challenges were not counterbalanced such that all children were presented with the sad film clips first, followed by the happy and fear clips. Of note, significant parasympathetic augmentation was observed from the neutral baseline film to the sad film, but not to the other two films. Likewise, only RSA reactivity in response to the sad film was related to prosocial behavior. Thus, in the absence of a counterbalanced design, it is impossible to rule out a plausible competing explanation for the obtained findings, which is that parasympathetic reactivity to any emotion challenge (or perhaps any negative emotion challenge) would be relevant for understanding prosocial behavior, but only the first film posed a significant regulatory challenge, perhaps because the intervening neutral film clips were not of sufficient duration to support full regulatory recovery to baseline levels. In future research, it will be important to test these hypotheses while counterbalancing the negative valence emotion elicitations around the happy stimulus presentation. Following Ray (2007), we advise against sequential pairing of negative emotion contexts in the absence of a positive emotion stimulus to minimize emotional fatigue.

Second, the use of films to elicit emotion states in this study constrained our capacity to understand how children may behave in real‐world situations, and may have curtailed the range of ANS reactivity expressed in this study. Although films are commonly used to elicit emotional responses with demonstrable ecological validity (Gross & Levenson, 1995; Kreibig, 2010; Kreibig, Wilhelm, Roth, & Gross, 2007; Ray, 2007; Uhrig et al., 2016), children may be more detached from passive film stimuli than from real‐life contexts eliciting these emotions. Moreover, in contrast to well‐validated emotion evocation tools, such as the Inventory of Affective Pictures (Lang, Bradley, & Cuthbert, 1997), the consistency of emotion challenges posed by film stimuli remains uncertain. Indeed, the content of each film may have been relatively more or less salient for subsets of children in the current sample. For example, the sad *Crooklyn* scene depicted a set of siblings learning about the death of their mother. Although most children in the current sample had not yet experienced the death of a primary caregiver, parental loss may have been experienced as a more realistic and relatable experience than the fanciful food fight scene in *Hook*, or the frightening scene depicting two boys playing on railroad tracks while a train quickly approached in *Stand by Me*. Likewise, the intensity of these film stimuli was limited (e.g., the two‐minute scene from *Crooklyn* depicted children crying after hearing about the death of their mother, rather than a scene of the mother actually dying) such that they were unlikely to evoke the kinds of extreme ANS reactivity responses that may be negatively associated with prosocial responding.

Third, the inclusion of an observational measure of prosocial donating behavior constitutes a major advance over prior studies, which have tended to rely on potentially biased self‐ or other‐reported prosociality, but the validity of the current laboratory‐observed donating behavior would have been enhanced by observations of children's prosocial behavior in real‐world contexts. Although we found the same, albeit marginal, positive quadratic relation between RSA reactivity to the sad film clip, but not to the happy or fear clips, and parent reports of prosocial behavior, it is noteworthy that observations of children's donating behavior were not significantly related to parent reports of children's prosocial behavior. Modest to moderate correlations across multiple informants and assessment modalities are not uncommon (Kraemer et al., 2003), and the replication of the observational model with parent reports strengthened our confidence in the observed effects. However, given that different prosocial expressions (e.g., sharing, helping, comforting) are largely orthogonal (Dunfield, Kuhlmeier, O'Connell, & Kelley, 2011), it is possible that parasympathetic regulation and/or responses to sadness are particularly salient for understanding prosocial sharing/donating, as opposed to other prosocial behaviors, which were included on the parent‐reported measure (e.g., is kind to younger children).

Fourth, the absence of an observational measure of prosocial donating behavior at age 7 limited our ability to render directional conclusions based on the observational findings. Although the marginal replication of these patterns with parent‐reported prosocial behavior over and above prior parent reports lends some support to the direction of effects, the optimal model would have included measures of all variables at all time points. That said, prior studies do point to a relatively high degree of stability in RSA reactivity during childhood (Calkins & Keane, 2004).

Fifth, although the current model controlled for the potential influence of gender, race/ethnicity, verbal ability, and SES on the obtained relations, additional covariates will be important to consider in future research. For example, future studies should consider the potential influence of children's emotion knowledge on patterns of parasympathetic reactivity to emotion‐eliciting films and/or in response to a prosocial donation prompt to assist critically ill children. Indeed, a wealth of empirical evidence suggests that emotion knowledge is integral to prosocial behavior because the actor must register the emotion cues of others, interpret them correctly, and act accordingly (for a review, see Denham, 1998).

Finally, the current investigation coded RSA using age‐adjusted respiratory frequency bands to account for children's higher rates of breathing (i.e., 0.15–0.8 Hz; Johnson et al., 2017). However, as noted by Shader et al. (2018), this range includes adult respiratory frequencies (i.e., those falling below 0.28 Hz), which may have introduced noise into our RSA calculations and underestimated children's parasympathetic reactivity patterns. As such, the current findings may have underestimated children's parasympathetic reactivity to the film stimuli. Importantly, Shader et al. (2018) focused on parasympathetic withdrawal, rather than augmentation; thus, it is not clear whether and how respiratory frequency bands may influence estimates of parasympathetic augmentation.

## 5 IMPLICATIONS FOR FUTURE RESEARCH AND PRACTICE

The current study points to complex relations between parasympathetic reactivity and prosocial behavior while illuminating future directions for research and practice. Specifically, the relation between ANS regulation and prosocial behavior may be more nuanced than suggested by prior linear evaluations, but also necessitates investigation across a range of emotional stimuli and intensities to capture the nature of this complexity fully. In light of recent interventions to promote prosocial behavior (e.g., Flook et al., 2015), these findings suggest that directing attention toward physiological processes may augment these efforts. Looking ahead, researchers must evaluate the dynamic role of ANS activity during all phases of regulation (i.e., rest, reactivity, and recovery) and in situations necessitating both extreme and moderate regulatory responses. Additional research is needed to examine ANS regulation in response to challenges that necessitate different types of regulation (i.e., augmentation vs. withdrawal), and as related to varied expressions of prosocial behavior (e.g., sharing, helping, and comforting).

The current findings suggest that flexible engagement with social stimuli promotes positive social development. This is consistent with clinical research and practice, which suggest that both excess and inhibited emotional responding may signal or precipitate pathological engagement with social stimuli (Perry, 1999; Siegel, 1999). Indeed, regulatory flexibility in response to environmental stressors is a central goal of several therapeutic intervention practices (e.g., mindfulness, cognitive behavioral therapy), because it connotes an ability to engage with difficulties and manage them, which eventuates in positive psychological outcomes (Bonanno & Burton, 2013; Gu, Strauss, Bond, & Cavanagh, 2015; Lloyd, Bond, & Flaxman, 2013). Future work should explore the degree to which a dynamic range of regulatory engagement with social stimuli is adaptive, as well as points at which it may become maladaptive.

## CONFLICT OF INTEREST

None declared.

## Data Availability

The data that support the findings of this study are available from the corresponding author upon reasonable request.

## References

[brb31380-bib-0001] Afifi, A. A. , Kotlerman, J. B. , Ettner, S. L. , & Cowan, M. (2007). Methods for improving regression analysis for skewed continuous or counted responses. Annual Review of Public Health, 28, 95–111. 10.1146/annurev.publhealth.28.082206.094100 17112339

[brb31380-bib-0002] Alkon, A. , Boyce, W. T. , Davis, N. V. , & Eskenazi, B. (2011). Developmental changes in autonomic nervous system resting and reactivity measures in Latino children from 6 to 60 months of age. Journal of Developmental & Behavioral Pediatrics, 32, 668–677. 10.1097/DBP.0b013e3182331fa6 22008788

[brb31380-bib-0003] Alkon, A. , Goldstein, L. H. , Smider, N. , Essex, M. J. , Kupfer, D. J. , & Boyce, W. T. (2003). Developmental and contextual influences on autonomic reactivity in young children. Developmental Psychobiology, 42(1), 64–78. 10.1002/dev.10082 12471637

[brb31380-bib-0004] Anastassiou‐Hadjicharalambous, X. , & Warden, D. (2008). Physiologically‐indexed and self‐perceived affective empathy in conduct‐disordered children high and low on callous‐unemotional traits. Child Psychiatry and Human Development, 39(4), 503 10.1007/s10578-008-0104-y 18792777

[brb31380-bib-0005] Bandstra, N. F. , Chambers, C. T. , McGrath, P. J. , & Moore, C. (2011). The behavioural expression of empathy to others' pain versus others' sadness in young children. Pain, 152(5), 1074–1082. 10.1016/j.pain.2011.01.024 21349641

[brb31380-bib-0006] Batson, C. D. , & Powell, A. A. (2003). Altruism and prosocial behavior In Millon T. & Lerner M. J. (Eds.), Handbook of psychology (vol. 5, pp. 463–484). Hoboken, NJ: John Wiley & Sons Inc.

[brb31380-bib-0007] Bauer, D. J. , & Curran, P. J. (2005). Probing interactions in fixed and multilevel regression: Inferential and graphical techniques. Multivariate Behavioral Research, 40(3), 373–400. 10.1207/s15327906mbr4003_5 26794689

[brb31380-bib-0008] Beauchaine, T. P. , Gatzke‐Kopp, L. , Neuhaus, E. , Chipman, J. , Reid, M. J. , & Webster‐Stratton, C. (2013). Sympathetic‐and parasympathetic‐linked cardiac function and prediction of externalizing behavior, emotion regulation, and prosocial behavior among preschoolers treated for ADHD. Journal of Consulting and Clinical Psychology, 81, 481–493. 10.1037/a0032302 23544677 PMC3952490

[brb31380-bib-0009] Benenson, J. F. , Pascoe, J. , & Radmore, N. (2007). Children's altruistic behavior in the dictator game. Evolution and Human Behavior, 28, 168–175. 10.1016/j.evolhumbehav.2006.10.003

[brb31380-bib-0010] Bennett, D. S. , & Lewis, M. (2011). [Children's emotional response to film stimuli]. Unpublished raw data.

[brb31380-bib-0011] Berntson, G. G. , Lozano, D. L. , Chen, Y. J. , & Cacioppo, J. T. (2004). Where to Q in PEP. Psychophysiology, 41(2), 333–337. 10.1111/j.1469-8986.2004.00156.x 15032999

[brb31380-bib-0012] Bonanno, G. A. , & Burton, C. L. (2013). Regulatory flexibility: An individual differences perspective on coping and emotion regulation. Perspectives on Psychological Science, 8(6), 591–612. 10.1177/1745691613504116 26173226

[brb31380-bib-0013] Burt, K. B. , & Obradović, J. (2013). The construct of psychophysiological reactivity: Statistical and psychometric issues. Developmental Review, 33(1), 29–57. 10.1016/j.dr.2012.10.002

[brb31380-bib-0014] Bush, N. R. , Alkon, A. , Obradović, J. , Stamperdahl, J. , & Boyce, W. T. (2011). Differentiating challenge reactivity from psychomotor activity in studies of children's psychophysiology: Considerations for theory and measurement. Journal of Experimental Child Psychology, 110(1), 62–79. 10.1016/j.jecp.2011.03.004 21524757 PMC4160114

[brb31380-bib-0015] Buss, K. A. , Davis, E. L. , Ram, N. , & Coccia, M. (2018). Dysregulated fear, social inhibition, and respiratory sinus arrhythmia: A replication and extension. Child Development, 89(3), e214–e228. 28326533 10.1111/cdev.12774 PMC5608616

[brb31380-bib-0016] Calkins, S. D. , & Fox, N. A. (2002). Self‐regulatory processes in early personality development: A multilevel approach to the study of childhood social withdrawal and aggression. Development and Psychopathology, 14, 477–498. 10.1017/S095457940200305X 12349870

[brb31380-bib-0017] Calkins, S. D. , & Keane, S. P. (2004). Cardiac vagal regulation across the preschool period: Stability, continuity, and implications for childhood adjustment. Developmental Psychobiology, 45(3), 101–112. 10.1002/dev.20020 15505799

[brb31380-bib-0018] Carlo, G. , Roesch, S. C. , Knight, G. P. , & Koller, S. H. (2001). Between‐or within‐culture variation? Culture group as a moderator of the relations between individual differences and resource allocation preferences. Journal of Applied Developmental Psychology, 22, 559–579. 10.1016/S0193-3973(01)00094-6

[brb31380-bib-0019] Clark, C. A. , Skowron, E. A. , Giuliano, R. J. , & Fisher, P. A. (2016). Intersections between cardiac physiology, emotion regulation and interpersonal warmth in preschoolers: Implications for drug abuse prevention from translational neuroscience. Drug and Alcohol Dependence, 163, S60–S69. 10.1016/j.drugalcdep.2016.01.033 27306733 PMC4911543

[brb31380-bib-0020] Cui, L. , Morris, A. S. , Harrist, A. W. , Larzelere, R. E. , Criss, M. M. , & Houltberg, B. J. (2015). Adolescent RSA responses during an anger discussion task: Relations to emotion regulation and adjustment. Emotion, 15, 360–372. 10.1037/emo0000040 25642723 PMC4437810

[brb31380-bib-0021] Davis, E. L. , Quiñones‐Camacho, L. E. , & Buss, K. A. (2016). The effects of distraction and reappraisal on children's parasympathetic regulation of sadness and fear. Journal of Experimental Child Psychology, 142, 344–358. 10.1016/j.jecp.2015.09.020 26601786 PMC4666770

[brb31380-bib-0022] Deci, E. L. (1996). Making room for self‐regulation: Some thoughts on the link between emotion and behavior. Psychological Inquiry, 7, 220–223. 10.1207/s15327965pli0703_3

[brb31380-bib-0023] Denham, S. A. (1998). Emotional development in young children. New York, NY: Guilford Press.

[brb31380-bib-0024] Diener, M. L. , & Kim, D.‐Y. (2004). Maternal and child predictors of preschool children's social competence. Journal of Applied Developmental Psychology, 25, 3–24. 10.1016/j.appdev.2003.11.006

[brb31380-bib-0025] Dunfield, K. , Kuhlmeier, V. A. , O'Connell, L. , & Kelley, E. (2011). Examining the diversity of prosocial behavior: Helping, sharing, and comforting in infancy. Infancy, 16, 227–247. 10.1111/j.1532-7078.2010.00041.x 32693496

[brb31380-bib-0026] Easterbrook, J. A. (1959). The effect of emotion on cue utilization and the organization of behavior. Psychological Review, 66, 183–200. 10.1037/h0047707 13658305

[brb31380-bib-0027] Eisenberg, N. (2010). Empathy‐related responding: Links with self‐regulation, moral judgment, and moral behavior In Mikulincer M. & Shaver P. R. (Eds.), Prosocial motives, emotions, and behavior: The better angels of our nature (pp. 129–148). Washington, DC: The American Psychological Association.

[brb31380-bib-0028] Eisenberg, N. , & Eggum, N. D. (2009). Empathic responding: Sympathy and personal distress. The Social Neuroscience of Empathy, 6, 71–83.

[brb31380-bib-0029] Eisenberg, N. , & Fabes, R. A. (1990). Empathy: Conceptualization, measurement, and relation to prosocial behavior. Motivation and Emotion, 14(2), 131–149. 10.1007/BF00991640

[brb31380-bib-0030] Eisenberg, N. , & Fabes, R. A. (1992). Emotion, regulation, and the development of social competence In Clark M. S. (Ed.), Review of personality and social psychology: Vol. 14. Emotion and social behavior (pp. 119–150).Thousand Oaks, CA: Sage Publications Inc.

[brb31380-bib-0031] Eisenberg, N. , Fabes, R. A. , Bustamante, D. , Mathy, R. M. , Miller, P. A. , & Lindholm, E. (1988). Differentiation of vicariously induced emotional reactions in children. Developmental Psychology, 24(2), 237 10.1037/0012-1649.24.2.237

[brb31380-bib-0032] Eisenberg, N. , Fabes, R. A. , Karbon, M. , Murphy, B. C. , Wosinski, M. , Polazzi, L. , … Juhnke, C. (1996). The relations of children's dispositional prosocial behavior to emotionality, regulation, and social functioning. Child Development, 67, 974–992. 10.2307/1131874 8706539

[brb31380-bib-0033] Eisenberg, N. , Fabes, R. A. , Miller, P. A. , Fultz, J. , Shell, R. , Mathy, R. M. , & Reno, R. R. (1989). Relation of sympathy and personal distress to prosocial behavior: A multimethod study. Journal of Personality and Social Psychology, 57, 55–66. 10.1037/0022-3514.57.1.55 2754604

[brb31380-bib-0034] Eisenberg, N. , Fabes, R. A. , Schaller, M. , Carlo, G. , & Miller, P. A. (1991). The relations of parental characteristics and practices to children's vicarious emotional responding. Child Development, 62(6), 1393–1408. 10.2307/1130814 1786723

[brb31380-bib-0035] Ekman, P. , Levenson, R. W. , & Friesen, W. V. (1983). Autonomic nervous system activity distinguishes among emotions. Science, 221(4616), 1208–1210. 6612338 10.1126/science.6612338

[brb31380-bib-0036] El‐Sheikh, M. , Harger, J. , & Whitson, S. M. (2001). Exposure to interparental conflict and children's adjustment and physical health: The moderating role of vagal tone. Child Development, 72(6), 1617–1636. 10.1111/1467-8624.00369 11768136

[brb31380-bib-0037] Fabes, R. A. , Eisenberg, N. , & Eisenbud, L. (1993). Behavioral and physiological correlates of children's reactions to others in distress. Developmental Psychology, 29(4), 655 10.1037/0012-1649.29.4.655

[brb31380-bib-0038] Flook, L. , Goldberg, S. B. , Pinger, L. , & Davidson, R. J. (2015). Promoting prosocial behavior and self‐regulatory skills in preschool children through a mindfulness‐based kindness curriculum. Developmental Psychology, 51, 44–51. 10.1037/a0038256 25383689 PMC4485612

[brb31380-bib-0039] Gill, K. L. , & Calkins, S. D. (2003). Do aggressive/destructive toddlers lack concern for others? Behavioral and physiological indicators of empathic responding in 2‐year‐old children. Development and Psychopathology, 15, 55–71. 10.1017/S095457940300004X 12848435

[brb31380-bib-0040] Goodman, R. (1997). The strengths and difficulties questionnaire: A research note. Journal of Child Psychology and Psychiatry, 38(5), 581–586. 10.1111/j.1469-7610.1997.tb01545.x 9255702

[brb31380-bib-0041] Gottlieb, G. , & Halpern, C. T. (2002). A relational view of causality in normal and abnormal development. Development and Psychopathology, 14(3), 421–435. 10.1017/S0954579402003024 12349867

[brb31380-bib-0042] Granger, D. A. , Kivlighan, K. T. , Fortunato, C. , Harmon, A. G. , Hibel, L. C. , Schwartz, E. B. , & Whembolua, G.‐L. (2007). Integration of salivary biomarkers into developmental and behaviorally‐oriented research: Problems and solutions for collecting specimens. Physiology & Behavior, 92(4), 583–590. 10.1016/j.physbeh.2007.05.004 17572453

[brb31380-bib-0043] Gross, J. J. (1998). The emerging field of emotion regulation: An integrative review. Review of General Psychology, 2(3), 271–299. 10.1037/1089-2680.2.3.271

[brb31380-bib-0044] Gross, J. J. (2015). Emotion regulation: Current status and future prospects. Psychological Inquiry, 26(1), 1–26. 10.1080/1047840X.2014.940781

[brb31380-bib-0045] Gross, J. J. , & Levenson, R. W. (1995). Emotion elicitation using films. Cognition & Emotion, 9(1), 87–108. 10.1080/02699939508408966

[brb31380-bib-0046] Grusec, J. E. , & Redler, E. (1980). Attribution, reinforcement, and altruism: A developmental analysis. Developmental Psychology, 16(5), 525–534. 10.1037/0012-1649.16.5.525

[brb31380-bib-0047] Gu, J. , Strauss, C. , Bond, R. , & Cavanagh, K. (2015). How do mindfulness‐based cognitive therapy and mindfulness‐based stress reduction improve mental health and wellbeing? A systematic review and meta‐analysis of mediation studies. Clinical Psychology Review, 37, 1–12. 10.1016/j.cpr.2015.01.006 25689576

[brb31380-bib-0048] Gunnar, M. R. , & Vazquez, D. (2015). Stress neurobiology and developmental psychopathology In Cicchetti D. (Ed.), Developmental psychopathology: Volume 2: Developmental neuroscience (pp. 533–577). Hoboken, NJ John Wiley and Sons Ltd.

[brb31380-bib-0049] Hastings, P. D. , Kahle, S. S. , & Han, G.‐H.‐P. (2014). Developmental affective psychophysiology: Using physiology to inform our understanding of emotional development. Children and Emotion, 26, 13–28.

[brb31380-bib-0050] Hastings, P. D. , & Miller, J. G. (2014). Autonomic regulation, polyvagal theory, and children's prosocial development In Padilla‐Walker L. & Carlo G. (Eds.), Prosocial development: A multidimensional approach (pp. 112–127). New York, NY: Oxford University Press.

[brb31380-bib-0051] Hastings, P. D. , Nuselovici, J. N. , Utendale, W. T. , Coutya, J. , McShane, K. E. , & Sullivan, C. (2008). Applying the polyvagal theory to children's emotion regulation: Social context, socialization, and adjustment. Biological Psychology, 79(3), 299–306. 10.1016/j.biopsycho.2008.07.005 18722499

[brb31380-bib-0052] Hastings, P. D. , Zahn‐Waxler, C. , Robinson, J. , Usher, B. , & Bridges, D. (2000). The development of concern for others in children with behavior problems. Developmental Psychology, 36(5), 531 10.1037/0012-1649.36.5.531 10976595

[brb31380-bib-0053] Hein, G. , Lamm, C. , Brodbeck, C. , & Singer, T. (2011). Skin conductance response to the pain of others predicts later costly helping. PLoS ONE, 6(8), e22759 10.1371/journal.pone.0022759 21826205 PMC3149614

[brb31380-bib-0054] Hinnant, J. B. , & El‐Sheikh, M. (2009). Children's externalizing and internalizing symptoms over time: The role of individual differences in patterns of RSA responding. Journal of Abnormal Child Psychology, 37(8), 1049 10.1007/s10802-009-9341-1 19711181

[brb31380-bib-0055] Hollingshead, A. B. (1975). Four factor index of social status.

[brb31380-bib-0056] Johnson, M. , Deardorff, J. , Davis, E. L. , Martinez, W. , Eskenazi, B. , & Alkon, A. (2017). The relationship between maternal responsivity, socioeconomic status, and resting autonomic nervous system functioning in Mexican American children. International Journal of Psychophysiology, 116, 45–52. 28238817 10.1016/j.ijpsycho.2017.02.010 PMC5446802

[brb31380-bib-0057] Johnson, P. O. , & Neyman, J. (1936). Tests of certain linear hypotheses and their application to some educational problems. Statistical Research Memoirs, 1, 57–93.

[brb31380-bib-0058] Kogan, A. , Oveis, C. , Carr, E. W. , Gruber, J. , Mauss, I. B. , Shallcross, A. , … Keltner, D. (2014). Vagal activity is quadratically related to prosocial traits, prosocial emotions, and observer perceptions of prosociality. Journal of Personality and Social Psychology, 107(6), 1051–1063. 10.1037/a0037509 25243414

[brb31380-bib-0059] Kraemer, H. C. , Measelle, J. R. , Ablow, J. C. , Essex, M. J. , Boyce, W. T. , & Kupfer, D. J. (2003). A new approach to integrating data from multiple informants in psychiatric assessment and research: Mixing and matching contexts and perspectives. American Journal of Psychiatry, 160(9), 1566–1577. 10.1176/appi.ajp.160.9.1566 12944328

[brb31380-bib-0060] Krantz, D. S. , & Manuck, S. B. (1984). Acute psychophysiologic reactivity and risk of cardiovascular disease: A review and methodologic critique. Psychological Bulletin, 96(3), 435 10.1037/0033-2909.96.3.435 6393178

[brb31380-bib-0061] Kreibig, S. D. (2010). Autonomic nervous system activity in emotion: A review. Biological Psychology, 84(3), 394–421. 10.1016/j.biopsycho.2010.03.010 20371374

[brb31380-bib-0062] Kreibig, S. D. , Wilhelm, F. H. , Roth, W. T. , & Gross, J. J. (2007). Cardiovascular, electrodermal, and respiratory response patterns to fear‐and sadness‐inducing films. Psychophysiology, 44(5), 787–806. 10.1111/j.1469-8986.2007.00550.x 17598878

[brb31380-bib-0063] Laird, R. D. , & De Los Reyes, A. (2013). Testing informant discrepancies as predictors of early adolescent psychopathology: Why difference scores cannot tell you what you want to know and how polynomial regression may. Journal of Abnormal Child Psychology, 41(1), 1–14. 10.1007/s10802-012-9659-y 22773360

[brb31380-bib-0064] Lang, P. J. , Bradley, M. M. , & Cuthbert, B. N. (1997). International affective picture system (IAPS): Technical manual and affective ratings. NIMH Center for the Study of Emotion and Attention, pp. 39–58.

[brb31380-bib-0065] Lench, H. C. , Flores, S. A. , & Bench, S. W. (2011). Discrete emotions predict changes in cognition, judgment, experience, behavior, and physiology: A meta‐analysis of experimental emotion elicitations. Psychological Bulletin, 137(5), 834–855. 10.1037/a0024244 21766999

[brb31380-bib-0066] Liew, J. , Eisenberg, N. , Spinrad, T. L. , Eggum, N. D. , Haugen, R. G. , Kupfer, A. , … Baham, M. E. (2011). Physiological regulation and fearfulness as predictors of young children's empathy‐related reactions. Social Development, 20(1), 111–134. 10.1111/j.1467-9507.2010.00575.x 22573929 PMC3346257

[brb31380-bib-0067] Lloyd, J. , Bond, F. W. , & Flaxman, P. E. (2013). The value of psychological flexibility: Examining psychological mechanisms underpinning a cognitive behavioural therapy intervention for burnout. Work & Stress, 27(2), 181–199. 10.1080/02678373.2013.782157

[brb31380-bib-0068] Manuck, S. B. , Kasprowicz, A. L. , & Muldoon, M. F. (1990). Behaviorally‐evoked cardiovascular reactivity and hypertension: Conceptual issues and potential associations. Annals of Behavioral Medicine, 12(1), 17–29. 10.1207/s15324796abm1201_2

[brb31380-bib-0069] Miles, S. B. , & Stipek, D. (2006). Contemporaneous and longitudinal associations between social behavior and literacy achievement in a sample of low‐income elementary school children. Child Development, 77(1), 103–117. 10.1111/j.1467-8624.2006.00859.x 16460528

[brb31380-bib-0070] Miller, J. G. (2018). Physiological mechanisms of prosociality. Current Opinion in Psychology, 20, 50–54. 10.1016/j.copsyc.2017.08.018 28837956

[brb31380-bib-0071] Miller, J. G. , Kahle, S. , & Hastings, P. D. (2015). Roots and benefits of costly giving: Young children's altruism is related to having less family wealth and more autonomic flexibility. Psychological Science, 26(7), 1038–1045. 10.1177/0956797615578476 26015412 PMC4504814

[brb31380-bib-0072] Miller, J. G. , Kahle, S. , & Hastings, P. D. (2017). Moderate baseline vagal tone predicts greater prosociality in children. Developmental Psychology, 53(2), 274–289. 10.1037/dev0000238 27819463 PMC5293607

[brb31380-bib-0073] Miller, J. G. , Nuselovici, J. N. , & Hastings, P. D. (2016). Nonrandom acts of kindness: Parasympathetic and subjective empathic responses to sadness predict children's prosociality. Child Development, 87(6), 1679–1690. 10.1111/cdev.12629 28262932 PMC5340080

[brb31380-bib-0074] Miller, J. , Stromeyer, W. R. , & Schwieterman, M. A. (2013). Extensions of the Johnson‐Neyman technique to linear models with curvilinear effects: Derivations and analytical tools. Multivariate Behavioral Research, 48(2), 267–300. 10.1080/00273171.2013.763567 26741727

[brb31380-bib-0075] Neuhaus, G. , Foorman, B. R. , Francis, D. J. , & Carlson, C. D. (2001). Measures of information processing in rapid automatized naming (RAN) and their relation to reading. Journal of Experimental Child Psychology, 78(4), 359–373. 10.1006/jecp.2000.2576 11243694

[brb31380-bib-0076] Padilla‐Walker, L. M. , & Christensen, K. J. (2011). Empathy and self‐regulation as mediators between parenting and adolescents' prosocial behavior toward strangers, friends, and family. Journal of Research on Adolescence, 21(3), 545–551. 10.1111/j.1532-7795.2010.00695.x 29144027

[brb31380-bib-0077] Perry, B. (1999). The memories of states: How the brain stores and retrieves traumatic experience In Goodwin J. M. & Attias R. (Eds.), Splintered reflections: Images of the body in trauma (pp. 9–39). New York, NY: Basic Books.

[brb31380-bib-0078] Porges, S. W. (1995). Orienting in a defensive world: Mammalian modifications of our evolutionary heritage. A Polyvagal Theory. Psychophysiology, 32(4), 301–318. 10.1111/j.1469-8986.1995.tb01213.x 7652107

[brb31380-bib-0079] Porges, S. W. (2007). The polyvagal perspective. Biological Psychology, 74(2), 116–143. 10.1016/j.biopsycho.2006.06.009 17049418 PMC1868418

[brb31380-bib-0080] Ray, R. D. (2007). Emotion elicitation using films In Coan J. A. & Allen J. J. B. (Eds.), Handbook of emotion elicitation and assessment (pp. 9–28). London, UK: Oxford University Press.

[brb31380-bib-0081] Rosseel, Y. (2012). Lavaan: An R package for structural equation modeling and more. Version 0.5–12 (BETA). Journal of Statistical Software, 48(2), 1–36.

[brb31380-bib-0082] Rudd, K. L. , & Yates, T. M. (2018). The implications of sympathetic and parasympathetic regulatory coordination for understanding child adjustment. Developmental Psychobiology, 60(8), 1023–1036. 10.1002/dev.21784 30370630

[brb31380-bib-0083] Rueda, M. R. , Posner, M. I. , & Rothbart, M. K. (2011). Attentional control and self‐regulation In Vohs K. D. & Baumeister R. F. (Eds.), Handbook of self‐regulation: Research, theory, and applications (2nd ed, pp. 284–299). New York, NY: The Guilford Press.

[brb31380-bib-0084] Scrimgeour, M. B. , Davis, E. L. , & Buss, K. A. (2016). You get what you get and you don't throw a fit!: Emotion socialization and child physiology jointly predict early prosocial development. Developmental Psychology, 52(1), 102–116. 10.1037/dev0000071 26569566 PMC4695310

[brb31380-bib-0085] Shader, T. M. , Gatzke-Kopp, L. M. , Crowell, S. E. , Reid, M. J. , Thayer, J. F. , Vasey, M. W. , … Beauchaine, T. P. (2018). Quantifying respiratory sinus arrhythmia: Effects of misspecifying breathing frequencies across development. Development and psychopathology, 30(1), 351–366. 28554343 10.1017/S0954579417000669

[brb31380-bib-0086] Siegel, D. J. (1999). The developing mind (Vol. 296). New York, NY: Guilford Press.

[brb31380-bib-0087] Skowron, E. A. , Cipriano‐Essel, E. , Gatzke‐Kopp, L. M. , Teti, D. M. , & Ammerman, R. T. (2014). Early adversity, RSA, and inhibitory control: Evidence of children's neurobiological sensitivity to social context. Developmental Psychobiology, 56(5), 964–978. 10.1002/dev.21175 24142832 PMC3992193

[brb31380-bib-0088] Sroufe, L. A. , & Rutter, M. (1984). The domain of developmental psychopathology. Child Development, 55(1), 17–29. 10.2307/1129832 6705619

[brb31380-bib-0089] Staton, L. , El‐Sheikh, M. , & Buckhalt, J. A. (2009). Respiratory sinus arrhythmia and cognitive functioning in children. Developmental Psychobiology, 51(3), 249–258. 10.1002/dev.20361 19107730

[brb31380-bib-0090] Suess, P. E. , Porges, S. W. , & Plude, D. J. (1994). Cardiac vagal tone and sustained attention in school‐age children. Psychophysiology, 31(1), 17–22. 10.1111/j.1469-8986.1994.tb01020.x 8146250

[brb31380-bib-0091] Thelen, E. , & Smith, L. B. (1998). Dynamic systems theories In Damon W. & Lerner R. M. (Eds.), Handbook of child psychology (pp. 563–634). New York, NY: Wiley & Sons.

[brb31380-bib-0092] Uhrig, M. K. , Trautmann, N. , Baumgärtner, U. , Treede, R.‐D. , Henrich, F. , Hiller, W. , & Marschall, S. (2016). Emotion elicitation: A comparison of pictures and films. Frontiers in Psychology, 7, 180 10.3389/fpsyg.2016.00180 26925007 PMC4756121

[brb31380-bib-0093] U.S. Census Bureau (2011). Current population survey: Annual social and economic suppolement. Retrieved from http://www.census.gov/population/hispanic/data/2011.html.

[brb31380-bib-0094] Veenstra, R. , Lindenberg, S. , Oldehinkel, A. J. , De Winter, A. F. , Verhulst, F. C. , & Ormel, J. (2008). Prosocial and antisocial behavior in preadolescence: Teachers' and parents' perceptions of the behavior of girls and boys. International Journal of Behavioral Development, 32(3), 243–251. 10.1177/0165025408089274

[brb31380-bib-0095] Vohs, K. D. , & Baumeister, R. F. (2016). Handbook of self‐regulation: Research, theory, and applications. New York, NY: Guilford Publications.

[brb31380-bib-0096] von Dawans, B. , Fischbacher, U. , Kirschbaum, C. , Fehr, E. , & Heinrichs, M. (2012). The social dimension of stress reactivity: Acute stress increases prosocial behavior in humans. Psychological Science, 23(6), 651–660. 10.1177/0956797611431576 22593119

[brb31380-bib-0097] Woodcock, R. W. , Mather, N. , & McGrew, K. S. (2001). Woodcock‐Johnson III tests of achievement (WJ‐III). Rolling Meadows, IL: Riverside Pub.

[brb31380-bib-0098] Zahn‐Waxler, C. , Cole, P. M. , Welsh, J. D. , & Fox, N. A. (1995). Psychophysiological correlates of empathy and prosocial behaviors in preschool children with behavior problems. Development and Psychopathology, 7(1), 27–48. 10.1017/S0954579400006325

[brb31380-bib-0099] Zhang, R. , & Wang, Z. (2019). The mediating effect of empathy in the quadratic relationship between children's resting RSA and sharing behavior. International Journal of Psychophysiology, 140, 8–14. 10.1016/j.ijpsycho.2019.03.012 30928668

